# Immunogenic Cell Death and Role of Nanomaterials Serving as Therapeutic Vaccine for Personalized Cancer Immunotherapy

**DOI:** 10.3389/fimmu.2022.925290

**Published:** 2022-06-30

**Authors:** Elena Catanzaro, Olivier Feron, André G. Skirtach, Dmitri V. Krysko

**Affiliations:** ^1^ Cell Death Investigation and Therapy (CDIT) Laboratory, Department of Human Structure and Repair, Ghent University, Ghent, Belgium; ^2^ Cancer Research Institute Ghent, Ghent, Belgium; ^3^ Cancer Translational Research Laboratory, Pole of Pharmacology and Therapeutics, Institut de Recherche Expérimentale et Clinique (IREC), UCLouvain, Brussels, Belgium; ^4^ Nano-BioTechnology Laboratory, Department of Biotechnology, Ghent University, Ghent, Belgium; ^5^ Institute of Biology and Biomedicine, National Research Lobachevsky State University of Nizhny Novgorod, Nizhny Novgorod, Russia; ^6^ Department of Pathophysiology, Sechenov First Moscow State Medical University (Sechenov University), Moscow, Russia

**Keywords:** ferroptosis apoptosis, necroptosis, pyroptosis, immunogenicity, immunogenic cell death, antitumor therapy, nanomaterials

## Abstract

Immunogenic cell death (ICD) is a rapidly growing research area representing one of the emerging therapeutic strategies of cancer immunotherapy. ICD is an umbrella term covering several cell death modalities including apoptosis, necroptosis, ferroptosis and pyroptosis, and is the product of a balanced combination of adjuvanticity (damage-associated molecular patterns and chemokines/cytokines) and antigenicity (tumor associated antigens). Only a limited number of anti-cancer therapies are available to induce ICD in experimental cancer therapies and even much less is available for clinical use. To overcome this limitation, nanomaterials can be used to increase the immunogenicity of cancer cells killed by anti-cancer therapy, which in themselves are not necessarily immunogenic. In this review, we outline the current state of knowledge of ICD modalities and discuss achievements in using nanomaterials to increase the immunogenicity of dying cancer cells. The emerging trends in modulating the immunogenicity of dying cancer cells in experimental and translational cancer therapies and the challenges facing them are described. In conclusion, nanomaterials are expected to drive further progress in their use to increase efficacy of anti-cancer therapy based on ICD induction and in the future, it is necessary to validate these strategies in clinical settings, which will be a challenging research area.

## Introduction

Cancer immunotherapy rearms the body’s immune system to fight cancer by mounting an effective anti-tumor immune response. Cancer immunotherapy has been recognized as the break-through technology of the year in 2013 (1). In 2018, the Nobel Prize in Physiology or Medicine was awarded to James Allison and Tasuku Honjo for their discovery of check-point inhibitors. Notably, one of the first attempts to treat cancer by activating the immune system goes back to 2600 BC, when the Egyptian pharaoh Imhotep, in order to induce infection and consequent inflammation, used poultice in combination with incision to treat cancer (2).

It is now well known that monotherapies and killing cancer cells simply by inducing apoptosis is not, by itself, particularly effective for treating cancer. Several anti-cancer approaches, including chemotherapy and radiotherapy need to be combined with immunotherapy to effectively induce activation of anti-tumor immunity leading to tumor eradication and generation of long-term immunity. Personalized immunotherapy is based on collecting tumor tissue by biopsy or during surgery and inducting cell death *in vitro* in the cells. After that, the cells are re-injected into the patient. This approach seems promising for raising the potency and tumor-specific immunity of cancer vaccines. The administration of cancer cell-based vaccines is particularly interesting because of the presence of all the cellular components, including tumor-associated antigens and ‘Damage-associated Molecular Patterns’ (DAMPs) released during cell death (3, 4) (**Box 1**). One of the emerging methods of immunotherapy involves the use of cancer cells undergoing immunogenic cell death (ICD) as a cell-based vaccine. ICD is an umbrella term covering several regulated cell death modalities. The immunogenic characteristics of ICD are mainly mediated by DAMPs and cytokines/chemokines (5–8). These molecules are intracellularly involved in fundamental biological processes and are normally not recognized by the immune system. But during ICD, these molecules are liberated into the extracellular environment, where they are recognized by corresponding pattern recognition receptors, leading to the activation of the innate and adaptive immune systems. The ICD concept has recently attracted special interest as a novel approach for personalized cancer immunotherapy.

Box 1 **Definition of immunogenicity and antigenicity of ICD. Immunogenicity and antigenicity are two major determinants of ICD. Immunogenicity of dying cancer cells is mediated mainly by DAMPs and/or cytokines/chemokines that are emitted from the dying cancer cells, they function as adjuvants and are key elements in the induction of cellular and humoral anti-tumor immune responses. By contrast, antigenicity of ICD refers to relevant antigens (*e.g.*, tumor-specific antigens, tumor-associated antigens or neoepitopes) which are revealed by the dying cancer cells, they account for specific recognition by antibodies generated as a result of the anti-tumor immune response. Both components need to be simultaneously present in dying cancer cells to efficiently support anti-tumor immunity.**

Anti-cancer vaccines based on dead cells should have at least two key features: the cancer cells must be able to efficiently undergo cell death by one of the known modalities, and the cell death must be immunogenic. In other words, the dying cells must be able to induce significant immunological anti-tumor responses. However, it is important to emphasize that one of the hallmarks of cancer cells is resistance to (apoptotic) cell death manifested in the acquisition of properties allowing them to circumvent or limit the apoptotic death pathways (9). To overcome apoptotic cell death resistance, anti-cancer therapies have to be designed to induce other ICD modalities. Indeed, it has been shown that cancer cells undergoing necroptosis (10, 11), ferroptosis (12) and pyroptosis (13, 14) are all immunogenic, fulfilling the first criterion for anti-cancer vaccines based on dead cancer cells, at least in experimental mouse models. However, it is important to stress that only limited anti-cancer therapies in clinical use can induce immunogenic forms of cell death. Inducers of different regulated cell death modalities (necroptosis, pyroptosis and ferroptosis) are available mostly for experimental cancer therapy. Therefore, one possible approach to overcome this limitation is to use nanomaterials to increase the immunogenicity of cancer cells killed by conventional anti-cancer therapy, which in themselves are not necessarily immunogenic (6).

**Figure 1:**
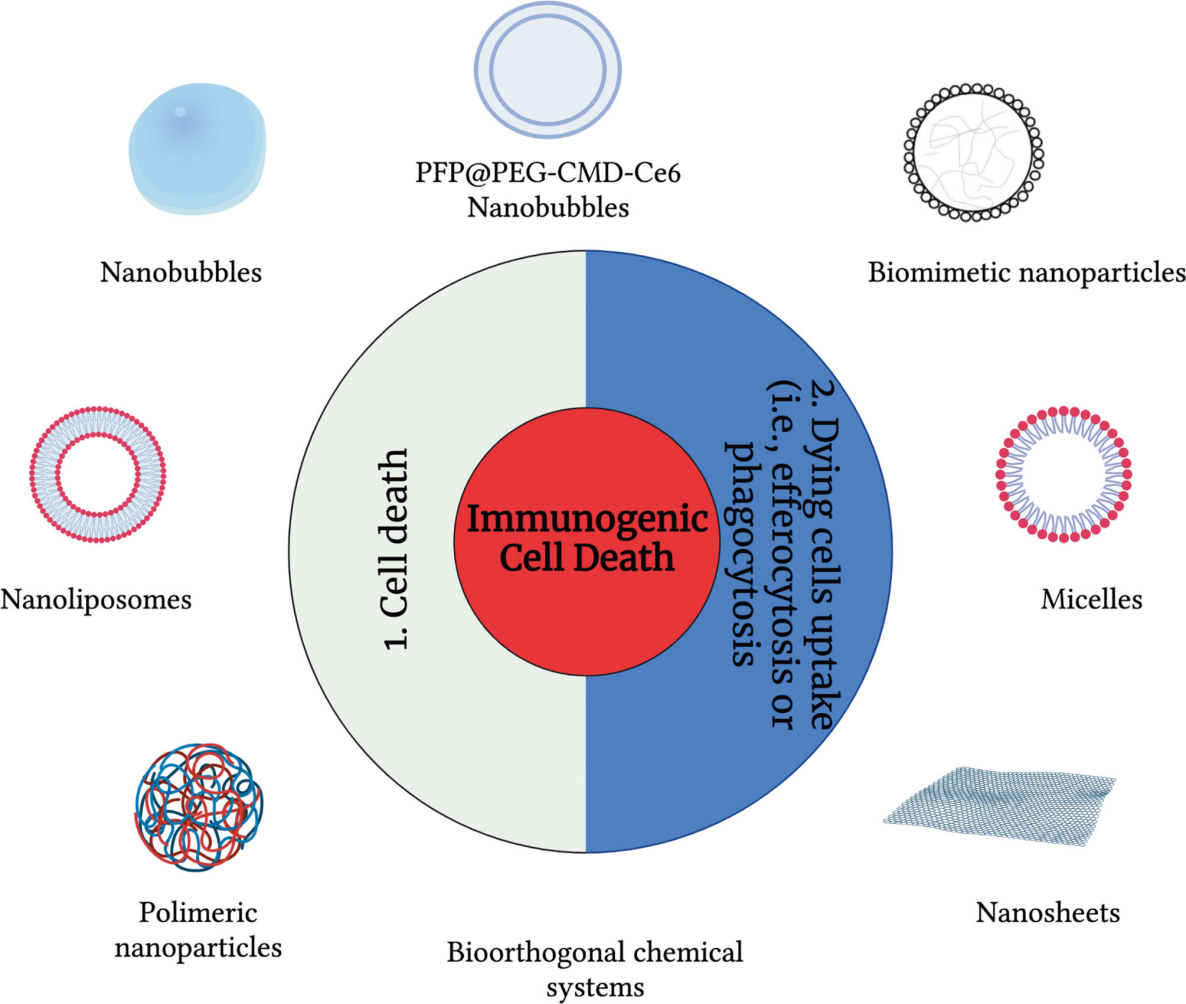
An overview of drug delivery carriers used to modulate immunogenic cell death modalities. To modulate the immunogenicity of cell death, drug delivery carriers can be used at three different levels: (1) cell death and (2) modulation of the dying cell’s uptake.

The use of a broad range of nanomaterials can help to address essential challenges and needs in controlling ICD and modulating the immunogenicity of cancer cell death modalities. Specifically, nanomaterials can be used to control the immunogenicity of cells (15), and they can also provide efficient, targeted delivery of immunomodulatory agents and their controlled release, which increases the efficiency of the treatment. Many nanomaterials, both organic-based and inorganic-based are available. In the context of cancer, nano-carriers were first applied to apoptosis. For over 20 years, drug delivery for apoptosis induction has stood out as a broad application area in which liposomes were among the first and most widely used carriers (**Figure 1**). Peculiarly, in the last ten years, almost all main classes of nanomaterials have been linked with immuno-oncology and immunogenicity and used to trigger apoptosis, including liposomes, micelles, dendrimers, organic nanoparticles (polymeric, hydrogel, lipid-based) and inorganic materials (metal, oxide, semiconductor, carbon) as well as special nanomaterials such as nanobubbles, cell penetrating peptides (CPP), antibodies, and exosomes (**Figure 1**). But since the discovery of other ICD modalities such as necroptosis, pyroptosis and ferroptosis, the use of nanomaterials to modulate the immunogenicity of cell death has been gaining pace.

In this review, we first give a short overview on the recent advances and the current state of knowledge of ICD modalities, including apoptosis, necroptosis, ferroptosis and pyroptosis, as well as on the achievements in the application of nanoparticles, nanomaterials and nano-drug delivery carriers in this area. In particular, since the role of nanomaterials in increasing immunogenicity of apoptosis is widely described elsewhere (16, 17), we will main focus our attention on the non-canonical cell deaths. Next, we will discuss potential mechanisms by which nanoparticle-based technology can be used to increase the immunogenicity of dying cancer cells based on the interplay between the use of nanomaterials and interference with the clearance of dying cancer cells. Finally, we will outline the challenges and opportunities and highlight the emerging trends in the modulation of the immunogenicity of dying cancer cells in experimental and translational cancer therapies.

## ICD Modalities at a Glance

Apoptosis is a physiological phenomenon that plays a crucial role in tissues development and cellular homeostasis. For instance, during organ development, tissue remodeling, or cellular replacement after injuries, the clearance of apoptotic cells by phagocytes (*e.g.*, macrophages) is crucial and allows the removal of dead cells without the induction of an immune- or -inflammatory response (18, 19). Indeed, in these settings apoptotic cells are silently recognized and removed by professional and non-professional phagocytes, in a process called efferocytosis. Efferocytosis happens before dying cells lose membrane integrity and avoids the pouring of the cellular content in the extracellular milieu. For this reason, for long time apoptotic cell death has been considered strictly immunologically silent (20, 21). These notions were supported by studies published in the early nineties by the group of Peter Henson and others. These researchers showed that the loss of phospholipid asymmetry in the plasma membrane of apoptotic cells leads to phosphatidylserine (PS) exposure on the outer surface of the lipid bilayer early during apoptosis, and that PS is responsible for recognition of those cells by antigen-presenting cells and for the immune-suppressive characteristics of apoptotic cell death (21, 22). During the same time, the “danger theory” of immunity was postulated by Polly Matzinger, who claimed that the immune system can distinguish self from non-self and dangerous signals from innocuous ones (23). This theory suggested that immune responses can be triggered by “*danger signals*” or DAMPs, which are released by the damaged/dying cells. It became clear that programmed cell death can be silent or tolerogenic depending on several factors, such as the initiating *stimuli* or the tumor microenvironment status (24). For instance, the lack of T cell infiltration, or the presence of immunosuppressive subtypes of engulfing cells, such as M2 macrophages, and the consequent production of immunosuppressive cytokines dictate an overall tolerogenic effect (25).

**Figure 2:**
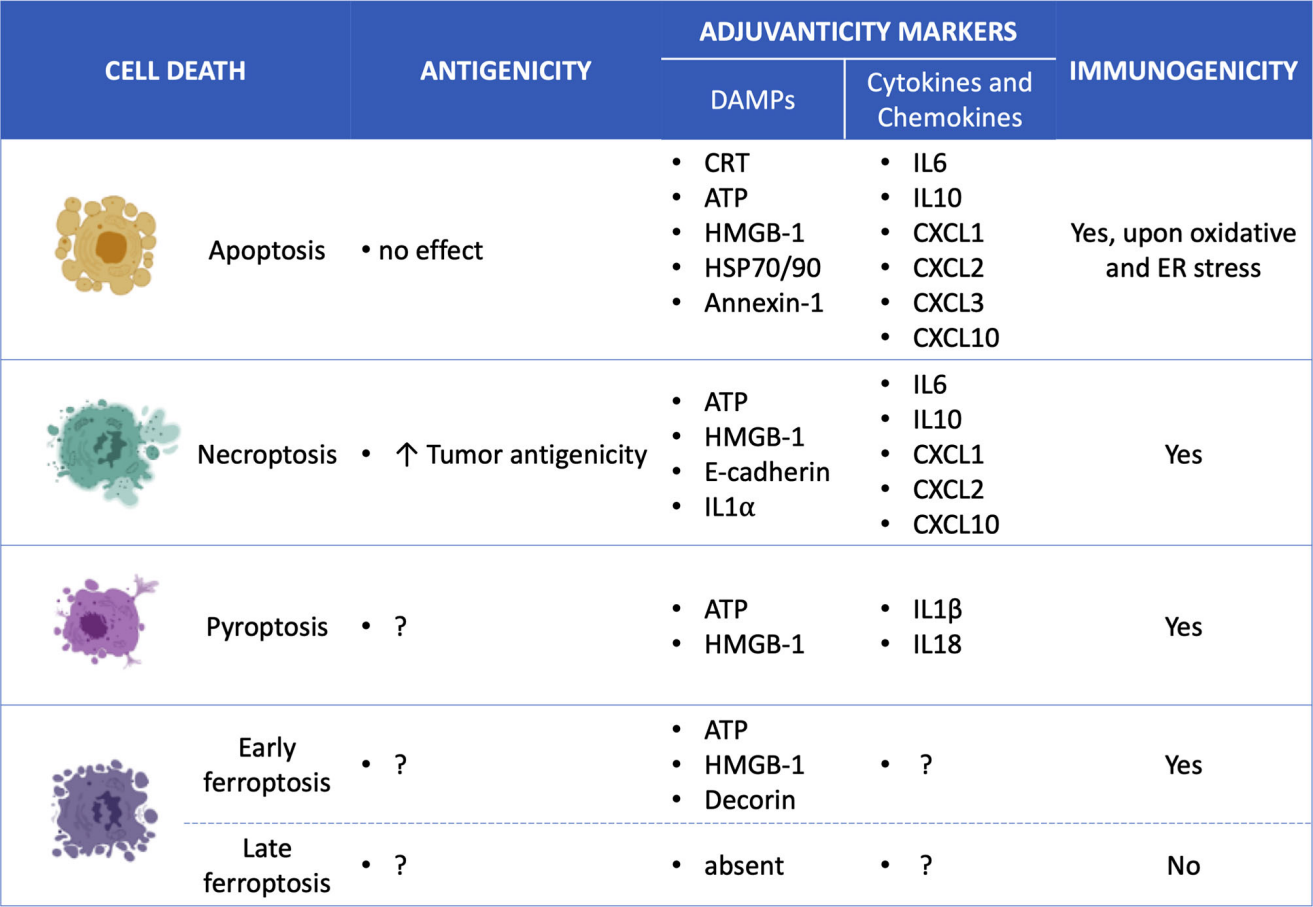
Overview of the specific ICD mediators for each cell death type. Apoptosis (7, 53, 54), Necroptosis (10, 53, 55, 56), Pyroptosis (14, 57–60); Ferroptosis (12) and; Damage-associated molecular patterns (DAMPs); Calreticulin (CRT); high mobility group box-1 (HMGB-1); interleukin (IL); chemokine (C-X-C motif) ligand (CXCL).

As opposite, a new concept of ICD emerged, initially referred to as immunogenic apoptosis (5, 26–29), because at that time apoptosis was the only known regulated form of cell death. Indeed, in the early 2000s, the dogma indicating a strict silent or tolerogenic apoptosis started to fall apart. AB1 mesothelioma cells expressing hemagglutinin primed CD8^+^ T cells when going through gemcitabine-mediated apoptosis elicited an effective immune response (30). Anthracyclines, such as doxorubicin, but not mitomycin c (26), and oxaliplatin, but not cisplatin (31), were able to initiate the immune system. It has been then demonstrated that apoptotic ICD is triggered only when cell death is caused by a concerted induction of oxidative stress and endoplasmic reticulum stress, which in turn, promote the activation of the emission of DAMPs (5). DAMPs act as find-me and/or eating-me signals for antigen-presenting cells mainly by interacting with pattern recognition receptors (PRRs). Then, the antigen presentation, together with the concurrent release of cytokine/chemokines trigger the polarization of interferon-γ (IFNγ)-producing CD8^+^ T cells and the activation of the adaptive immune response (5, 32) (**Box 1**). Later it has become clear that many other non-apoptotic cell death modalities can also be regulated and experimentally triggered in cancer cells. In this section, we will give a bird’s eye view of the main non-apoptotic cell death modalities (necroptosis, pyroptosis and ferroptosis) with focus on recent advances supporting their immunogenicity (**Figure 2**). Interestingly, for all types of cell death, certain nanomaterials favor the onset of immune system activation and improves the efficiency of cell-death induced anti-tumor activity (33–45).

### Necroptosis

Necroptosis is one of the best characterized cell death modalities. It is mediated by the kinase activities of receptor interacting protein kinase-1 (RIPK1) and RIPK3 and activation of the executioner protein mixed lineage kinase domain-like (MLKL), leading to rapid membrane permeabilisation and release of intracellular contents into the extracellular environment (46–50). Although it was initially shown that necroptotic cells do not induce pro-inflammatory responses (51, 52), several recent reports indicate that necroptotic cancer cells are immunogenic (**Figure 2**) and can induce effective anti-tumor immune responses (55, 61). It has been shown that necroptotic cells are engulfed by macropinocytosis which is characterized by formation of specious macropinosomes and co-ingestion of extracellular fluid (62, 63). When necroptotic cancer cells are used as a vaccine to protect mice against subsequent challenge with viable cancer cells, they release DAMPs such as HMGB1 and ATP and can induce activation and maturation of bone-marrow derived dendritic cells *in vitro* and *in vivo* (61). In that study, the authors showed that necroptotic cancer cells induce cross-priming of T cells *in vivo*. Also interesting is another study showing that necroptotic cancer cells can induce the release of pro-inflammatory cytokines and chemokines in addition to DAMPs (55). It has been demonstrated that transcriptional signaling downstream of RIPK1/RIPK3/NF-κB activation results in necroptotic cells producing pro-inflammatory cytokines/chemokines, which is a key determinant for the cross-priming of CD8^+^ T cells and critical for the immunogenicity of necroptotic cancer cells (55). A subsequent study clarified the mechanism by which effector immune cells interact with necroptotic cells (64). Intra-tumoral injection of necroptotic cells promotes BATF3^+^ cDC1^-^ and CD8^+^ leukocyte-dependent anti-tumor immunity accompanied by increased tumor antigen loading by tumor-associated antigen-presenting cells. That study further confirmed an initial observation (55) that immune-mediated tumor control by necroptotic cells requires NF-κB activation within necroptotic cells but not MLKL-mediated and cell lysis-dependent DAMP release (64).

**Figure 3:**
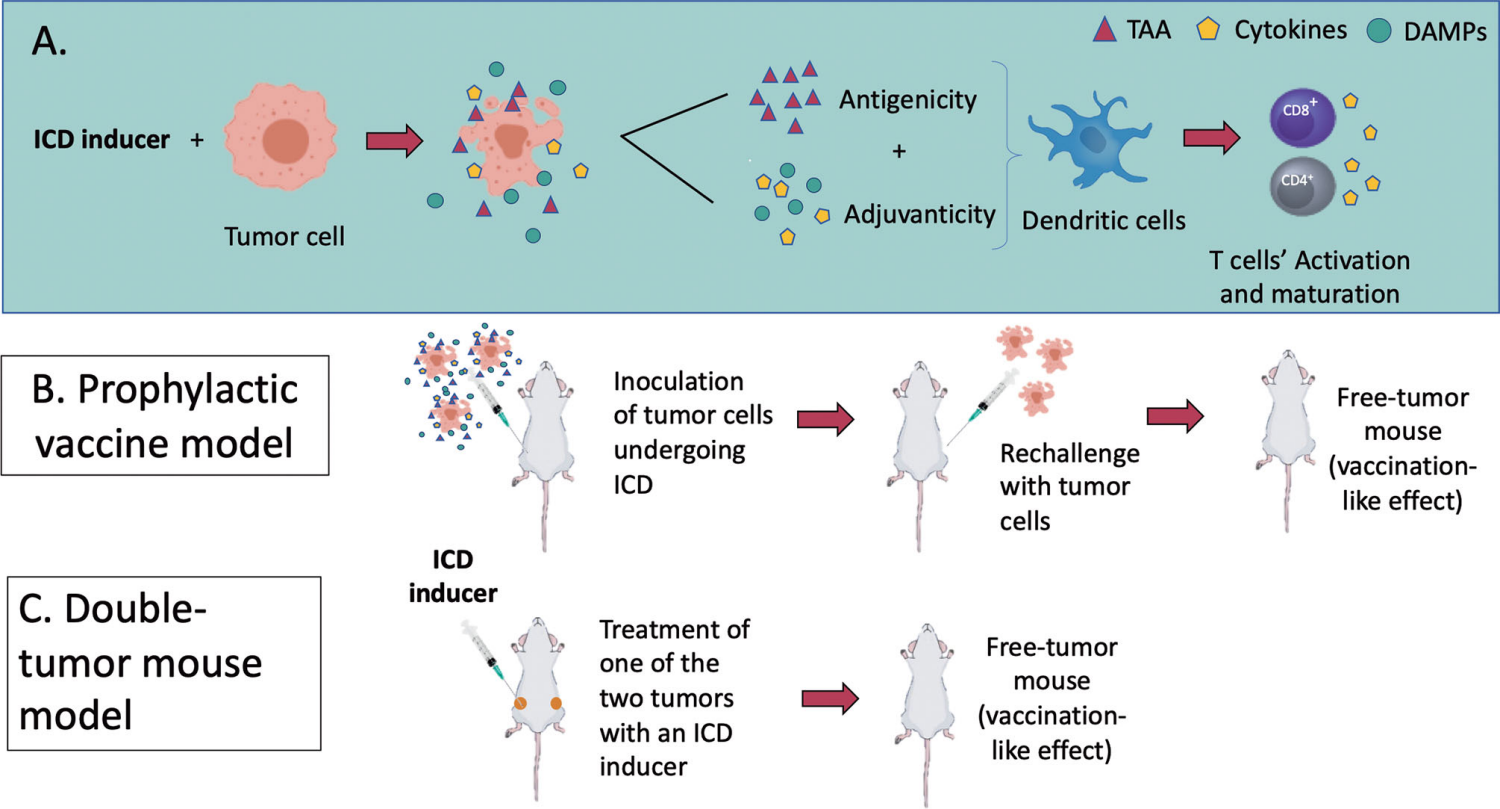
**(A)** An overview of the molecular mechanisms of ICD modalities and the *in vivo* models to assess it. ICD results as a balanced combination of adjuvanticity (induced by DAMPs and chemokines/cytokines) and antigenicity (tumour-associated antigens), which together promote the recruitment of antigen-presenting cells (APCs) and stimulate their ability to take up particulate material and cross-present dead cell-associated antigens to CD4^+^ and CD8^+^ cytotoxic T lymphocytes (**Box 1**). (**B**, **C**) represent the two recognized models to assess ICD *in vivo*. **(B)** In a tumour prophylactic vaccination model, cancer cells are first induced *in vitro* to undergo a particular form of ICD (apoptosis, necroptosis, pyroptosis or ferroptosis). They are then inoculated subcutaneously on one flank of the mice. Eight days later, the mice are challenged subcutaneously on the opposite flank with live cancer cells. Tumour growth at the challenge site is monitored. **(C)** In the bilateral tumour model, only the primary tumour is treated with the ICD inducers. The ability of mice to reject or limit the growth of primary and distant tumours is considered a sign of protective anticancer immunity.

However, the role of NF-kB activation in necroptosis-induced activation of antitumor immune responses is still under discussion. It has been shown that the immunogenicity of necroptotic tumor cells is not correlated with the NF-κB activation status (56, 61). But what is more interesting in the mechanisms of necroptotic immunogenicity is the antigenic properties of necroptotic cancer cells. In fact, the ability of dying cancer cells to induce anti-cancer immunity requires both antigenicity and adjuvanticity (65, 66). Adjuvanticity is mediated by the release of DAMPs and pro-inflammatory cytokines/chemokines from the dying cancer cells, which stimulate corresponding pattern recognition receptors of the innate immune system (**Figure 3**). However, the presence of these adjuvant signals is not sufficient to elicit an effective immune response against cancer cells. The cancer cells should also have strong antigenic properties, including tumor associated antigens (TAA), which can trigger an adaptive immune response under specific conditions in the presence of adjuvants (*i.e*., DAMPs) (**Box 1**). A recent study highlighted this hidden aspect of the immunogenicity of necroptotic cancer cells (**Figures 2**, **3**), *i.e*., their antigenicity, and underlined the tight correlation between the cell death type that cancer cells undergo and their antigenicity (56). That study showed in a murine prophylactic tumor vaccination model that when immune-dominant tumor antigen (AH1) was knocked out, necroptotic cancer cells were more immunogenic than apoptotic cells (56). That work suggests that the cell death modality may determine the strength of the immune response to alternative neoepitopes and that necroptosis is probably preferable to apoptosis in the treatment of cancer: necroptotic cancer cells can give rise to a broader antitumor immune response against tumor-intrinsic neoepitopes. Identification of the effects of different cell death modalities on the pattern of antigenic epitopes can become a highly valuable tool for future cancer immunotherapy.

Necroptosis can be also employed to modulate the immunogenicity of nanomaterials (**Figure 1**). For instance, necroptotic cell death contributes to the immunogenicity of near-infrared (NIR) responsive nanomaterials in combination with photoimmunotherapy (PTT). NIR-responsive nanomaterials have been shown to induce intratumor necroptosis that translates into the activation of both humoral and cell-mediated immune systems and photothermal tumor ablation. Black phosphorous nanosheets (BPs) excited with a specific laser is a vivid example of this. When injected into tumor-bearing mice, BPs localize in tumors, and their activation with laser promotes necroptosis, which triggers the immune response. On 4T1 breast cancer cells, BP-PTT modulates the most crucial markers of ICD, namely, exposure of calreticulin (CRT) and release of high mobility group box-1 (HMGB-1) and ATP. In addition, medium obtained from BP-PTT 4T1-treated cells, promoted macrophages to secrete tumor necrosis factor-α (TNF-α) and interleukin 6 (IL-6) which have positive immune stimulatory properties. *In vivo* studies confirmed that BP-PTT activates a comprehensive humoral and cellular immune response. 4T1 cells were injected in the left and right dorsal flank of Balb/c mice and one of them was irradiated with the NIR laser. The necroptosis occurring on one of the two tumor masses was sufficient to suppress tumor growth on both xenografts, which indicates systemic immune activation. Indeed, BP-PTT promoted the activation of both CD8^+^ and CD4^+^ T cells, together with downregulation of regulatory T cells, which are involved in negative immune regulation. Also, given the ease of structural manipulation of nanomaterials to further increase their immunogenicity, BP was conjugated with the adjuvant CpG to create a BPCP nanocomposite, significantly increasing BP immune stimulation (**Table 1**). Notably, no toxicity was observed, suggesting that BP nanomaterial is highly compatible as an antitumor and immunostimulant agent (67).

**Table 1:** Pre-clinical application of nanomaterials for cancer immunotherapy based on ICD.

Material	Application (therapy)	Experimental conditions	Therapeutic agents/adjuvant	Tumor model	Cell death type	Effect	Refs
Black phosphorus nanosheets	PTT	60 µg/mL + 808 nm, 2W/cm^2^, 180 s	CpG	4T1	Necroptosis	CRT activation	(67)
						HMGB-1 release	
						ATP secretion	
				Raw 264.7		↑ Phagocytosis potential	
						↑ TNFα	
						↑ IL-6	
		2 mg/Kg + 808 nm, 2W/cm^2^, 1 min		Bilateral 4T1 xenografted Balb/c mice		Primary and secondary tumor regression	
						CD8^+^ T cells activation	
						CD4^+^ T cells activation	
						↓ Treg cells	
						↑ TNFα	
						↑ IL-2	
						↑ IFNɣ	
PFP@PEG-CMD-Ce6 Nanobubbles	US	100 µg/mL + 300 s, power: 30 W, duty cycle: 20%, pulse repetition frequency: 1Hz, Y interval: 1mm	/	CT26	RIPK3/MLKL independent necroptosis and apoptosis	HMGB-1 release	(68)
				BMDCs		↑ CD86^+^	
		On days 8, 11, 14, 1-3 mg Ce6/kg doses + time: 600 sec, power: 10 W, duty cycle: 20%, pulse repetition frequency: 1 Hz, Y interval: 1 mm		CT26 xenografted Balb/c mice		↓ Tumor growth	
			PD-L1 100 µg/mouse			↓↓ Tumor growth	
						↓ Metastasis	
						↑ CD8^+^ T cells infiltration	
Poly(lactic-co-glycolic acid) polymeric core-bearing breast cancer membrane biomimetic nanoparticle (BN)	NIR-photoactivation	808 nm, 0.5 W/cm^2^, 2 min	Indocyanine green (ICG) 40 µg/mL + decitabine (DCT) 7.6 µg/mL	4T1	Pyroptosis and apoptosis	↑ Ca^2+^	(58)
						↑ Cyt c	
						↑ caspase 3	
						↑ GSDME	
						↑ N-terminal GSDME	
				(4T1 + BN) + BMDCs		↑ CD86^+^	
						↑ TNFα	
						↑ IL-6	
			ICG 1 mg/kg + DCT 0.19 mg/kg	4T1 xenografted Balb/c mice		↑ caspase 3	
						↑ N-terminal GSDME	
				Bilateral 4T1 xenografted Balb/c mice		Primary and secondary tumor regression	
						↑ TNFα	
						↑ IL-6	
						↑ IFNɣ	
						↑ DCs infiltration	
						↑ CD8^+^ T cells infiltration	
						↑ CD4+ T cells infiltration	
						↓ Treg cells	
Nanoliposome	/	/	DAC pre-treatment (100 µL) + cisplatinum (80 µmol/L^-1^)	4T1 xenografted Balb/c mice	Pyroptosis and apoptosis	↓ Tumor growth	(69)
						↓ Metastasis	
						↑ CD8^+^ T cells infiltration	
						↑ DCs maturation	
Arginine-rich manganese silicate nanobubbles	/	1 - 25 µg/mL	/	Huh7	Ferroptosis	↓ GSH; ↑ GSSG	(70)
						↓ GPX4	
		5 mg/kg		Huh7 xenografted Balb/c mice		↓ Tumor growth	
PEGylated single-atom Fe-containing nanocatalysts	/	25 - 400 µg/mL	/	4T1	Ferroptosis and apoptosis	/	(43)
		20 mg/kg		4T1 xenografted Balb/c mice		↓ Tumor growth	
	NIR-photoactivation	20 mg/kg; 808 nm, 1.5 W/cm^2^, 5 min				Complete tumor regression	
Fe-doxorubicin preloaded amorphous CaCo3 nanoformulation	/	/	Doxorubicin 5 mg/kg + Fe^2+^	4T1 and A375 nude mice	Ferroptosis and apoptosis	↓ Tumor growth No toxicity	(71)

↑ means "increase"; ↓ means "decrease".

Nanobubbles (NBs) exposed to ultrasound represent another nanoparticle model for inducing immunogenic necroptosis. Acoustic cavitation generated by ultrasound in chlorine e 6 PEGylated carboxymethyl dextran (PEG-CMD-Ce6) nanobubbles triggers an intense RIPK3-independent necroptosis coupled with immunostimulant effects. In CT26 cells, the stimulated NBs promote HMGB1 release and DC maturation. In Balb/c mice, after systemic administration of NBs, they localize mainly in the tumor and limit tumor growth, especially when they are administered in combination with the immune check-point inhibitor PD-L1. Indeed, combining ultrasound-NBs-mediated necroptosis with PD-L1 considerably strengthened the immune response in terms of the number of CD8^+^ T lymphocytes entering into the tumor bed and the prevention of metastatic spread (68) (**Table 1**). What emerges from these studies is that the advantages of nanoparticles, such as tumor localization and the ability to conjugate them with different active molecules, is that they represent a safe and effective antitumor immunotherapy that induces necroptosis.

### Pyroptosis

Pyroptosis is a proinflammatory form of regulated cell death triggered in response to pathogenic infections. It is caspase-dependent and mediated by pore-forming proteins named gasdermins (GSDMs) (13), but its role in cancer is still under investigation. Although it is a proinflammatory cell-death modality and could promote tumor progression, there is little evidence that induction of pyroptosis in cells undergoing carcinogenesis can suppress tumors (47, 72, 73). Nevertheless, pyroptosis is being explored as a new antitumor strategy to enhance antitumor immunity and overcome resistance to apoptosis (14, 47, 57, 74).

Morphologically, pyroptosis is characterized by DNA fragmentation and chromatin condensation accompanied by cellular swelling and bubble formation. The development of transmembrane pores caused by GSDM cleavage provokes the release of inflammatory cytokines and DAMPs and irreversible disruption of the osmotic equilibrium, which leads to cell membrane rupture (75, 76). Proinflammatory caspases such as caspases 1 and 11 and proapoptotic caspase 3 trigger different but intertwined pyroptotic pathways converging on the activation of GSDMs. The canonical pyroptotic pathway involves caspase 1 and is characterized by the formation of inflammasomes, while caspase 11 (the mouse orthologue of human caspases 4 and 5) and caspase 3 activate two non-canonical pathways directly targeting the effector protein (GSDMs), which are activated by cleavage into two fragments. The N-terminal fragment is involved in the formation of transmembrane pores linking the cytosol with the extracellular environment. The consequent potassium efflux and water influx alter the membrane potential and cellular homeostasis, causing cell rupture. Also, through the canonical pathway, caspase 1 activates the inflammatory cytokines pro-IL-1β and pro-IL-18, which are released through the pores and initiate the inflammatory response.

Given the pro-inflammatory nature of pyroptosis and the release of DAMPs and cytokines, this kind of cell death is highly immunogenic (**Figure 2**). Interestingly, the anticancer and immunostimulant activities are interconnected and interdependent. An intact and functioning immune system is required for pyroptotic tumor eradication, and the activation of GSDMs fosters the stimulation of both innate and adaptive tumor immunity (13, 14, 57). For instance, GSDME activation suppresses tumor growth by increasing the antitumor properties of tumor-infiltrating NK cells and CD4^+^ and CD8^+^ T lymphocytes (57). Only recently, it has been shown that the apoptosis effector caspase 3 can also promote the cleavage of GSDMs in cells with high levels of GSDMs (13, 47, 57). Thus, due to the connection between GSDMs and the apoptotic pathway, the activation of these proteins allows the conversion of non-inflammatory cell death (*i.e*. apoptosis) to inflammatory and immunogenic pyroptosis (13, 14, 57). The coexistence of apoptosis and pyroptosis could be considered as a new and noteworthy strategy to counteract apoptosis resistance, increase the chances of positive therapeutic outcomes, and, due to stimulation of the adaptive immune system, limit the incidence of metastasis and relapse. Of interest, the induction of mixed cell death types may provide a beneficial strategy to overcome the development of cancer resistance to a particular cell death type (29), increasing the chances for efficient cancer cell death killing.

However, GSDMs levels are often low in many tumors due to, for example, hypermethylation of mRNA (58, 69, 77). This notion together with the fact that pyroptosis can be used for antitumor therapy has prompted different research groups to exploit the efficiency and versatility of nanoparticles to directly increase GSDM levels in different tumor models to induce caspase-3 mediated pyroptosis (**Figure 1**). Zhao *et al.* (58) and Fan *et al.* (69) recently approached the problem by targeting the DFNA5 gene (which is responsible for hypermethylation of GSDME genes) with the methyltransferase inhibitor, decitabine (DAC) (69, 77). Fan *et al.* (69) loaded tumour-targeted nanoliposomes with the apoptotic inducer, cisplatin, and pre-treated tumours with DAC. The increased levels of GSDME enabled the nanoliposomes to trigger both apoptosis and pyroptosis, boosting cisplatin’s overall antitumor potential and remarkably stimulating the immune system. The nanoliposomes counteracted tumor growth and prevented metastasis in Balb/c mice bearing 4T1 tumors, while substantially increasing DC maturation in the lymph nodes. It also induced the transformation of native CD8^+^ T lymphocytes into central memory CD8^+^ cells in the spleen, showing that it increased the systemic immune response compared to the effect of cisplatin in the absence of DAC (69) (**Table 1**).

Using the same strategy, Zhao *et al.* (58) used a polymer-based nano-formulation to deliver both DAC and the proapoptotic indocyanine green to tumors. In this case, NIR sensitive biomimetic nanoparticles (BNP) were chosen to contain the two chemotherapeutic agents. Photoactivation of BNP promoted both apoptosis and pyroptosis (**Figure 1**). *In vitro*, BNP-photodynamic therapy induced the maturation of DCs and the secretion of IL-6 and TNF-α. *In vivo*, in a double xenograft model, photoactivating BNP prevented the growth of both irradiated and non-irradiated neoplastic lesions and induced a significantly larger amount of both CD4^+^ and CD8^+^ T cells in the spleen and in the non-irradiated tumor, together with active secretion of IFN-ɣ (58). It is notable that both nanomaterial constructs were well tolerated in the animal models, highlighting the high potential of this therapeutic strategy (58, 69).

Alternatively, instead of inducing the expression of GSDMs in cells, GSDMs can be transported into the tumor. Wang and colleagues created an elegant biorthogonal system to bring GSDMA3 into tumors and release it in a controlled way. They created an *ortho*-carbamoylmethylene silyl-phenolic ether system as a vehicle and attached to it GSDMA3 for delivery inside tumor cells. There, phenylalanine trifluoroborate (Phe-BF3) was used to release GSDMA3 from the system through a desilylation reaction. This system’s ability to induce pyroptosis was not astonishing (less than 15% of pyroptotic cells), but astonishing was that this small scale of cell death was sufficient to clear the entire 4T1 xenograft in Balb/c mice and to efficiently activate adaptive immunity. Notably, three administrations of nanoparticle-mediated delivery of GSDMA3 combined with Phe-BF3 were necessary to eradicate tumors, but just one round was effective when it was used together with PD-L1. In both situations, infiltration by NK cells and CD8^+^ and CD4^+^ T cells increased, while the levels of CD4^+^FOXP3^+^ T regulatory cells decreased. In parallel, the same treatment increased the M1 macrophage population whereas decreased the immunosuppressive M2 macrophage population (14). These results suggest that activation of GSDMs is an interesting strategy to increase the antitumor immunity-stimulating potential of silent cell death and that application of nanomaterials is an effective and safe method to achieve that (**Figure 2**).

Nonetheless, pyroptosis has not always proven to be advantageous for all the immunotherapy modalities. It has been shown that T cells with a CD19 chimeric antigen receptor against B cell malignancies trigger intense caspase 3/GSDME-dependent pyroptosis in targeted cells *via* granzyme B release. GSDME induces positive feedback resulting in the activation of the canonical pyroptotic pathway in macrophages, leading to a cytokine storm, which is a dangerous adverse reaction consisting of fever, hypotension, and respiratory insufficiency (78–80). In addition, pyroptosis is characterized by the active release of IL-1β, which is hyperinflammatory and could trigger detrimental effects in the long run, such as autoimmune diseases and cancer initiation and progression (81, 82). So far, the use of biomaterials seems to be useful for preventing pyroptosis resistance in cancer cells and increasing the overall immunotherapeutic potential of pyroptotic cancer cells. But as no chronic toxicity studies have been done, chronic adverse reactions cannot be excluded. Thus, future studies elucidating the mechanisms involved in the different pyroptosis pathways will enhance our understanding of the potential of pyroptosis as an anticancer strategy and of the use of pyroptosis to increase anticancer immunity, as well as delineating the toxicological profile.

### Ferroptosis

Ferroptosis is another regulated form of cell death recently recognized for its effectiveness in cancer therapy (83). Ferroptosis execution is characterized by an iron-catalyzed excessive peroxidation of polyunsaturated fatty acids (PUFAs). Consecutive depletion of PUFAs from plasma membrane alters membrane fluidity and structure, thereby increasing membrane permeability and altering integrity (84, 85). By using atomic force microscopy we have also shown that ferroptosis is accompanied by a decrease in overall elasticity of the ferroptotic cells which distinguishes it from apoptosis (50). In addition, lipid hydroperoxides may decompose to reactive toxic aldehydes such as malondialdehydes (MDA) or 4-hydroxy-2-nonenals (4-HNE), which upon crosslinking may inactivate key membrane proteins and promote ferroptosis. Damages resulting from lipid peroxidation can also occur in other subcellular locations. Among them, mitochondria may undergo severe stress causing loss of function and cytological changes, such as shrinkage and reduction in mitochondrial cristae (86, 87).

Free PUFA or PUFA-containing phospholipids are oxidized through non-enzymatic oxidation by free radicals or enzymatic activity of lipoxygenases (LOX), cyclooxygenases (COX) or cytochrome P450 oxidoreductases (88–90). Moreover, polyunsaturated ether phospholipids act as substrates for lipid peroxidation, contributing to the ferroptosis induction (91). Among the various classes of phospholipids, specific oxidation of phosphatidylethanolamines is proposed to give rise to unique lipid death signals that drive the execution of ferroptosis (89). Still, regardless of the enzymatic or non-enzymatic modes of lipid peroxidation, the canonical ferroptotic pathway requires the inactivation of mechanisms protecting cells against peroxidation damage (50, 84, 85, 89, 92–94) and/or the presence of excess cellular PUFAs. The major detoxifying mechanism involves the activity of glutathione peroxidases (GPX). GPX4, the major GPX enzyme capable to detoxify hydroperoxides in lipids inserted into membranes, is inactivated upon depletion of intracellular glutathione (GSH) which may itself result from the blockade of the x_c_ cystine/glutamate antiporter system. As for the excess PUFAs, it may result from an enhanced PUFA uptake or deficit in PUFA storage into lipid droplets that is thought to protect PUFA from peroxidation (94). We recently documented that cancer cells located in the acidic compartment of tumors exhibit a shift from glucose to lipid metabolism (95) and interestingly, that the consecutive stimulated uptake of FA represents a vulnerability for these cancer cells that undergo ferroptosis in response to an increase in dietary PUFAs (94). These data together with the higher oxidative stress usually observed in cancer cells contribute to make drugs inducing ferroptosis a valuable alternative to more conventional anticancer therapies leading to apoptosis or necroptosis.

Though many findings hint at the immunogenicity of ferroptosis, however, its immune-stimulating potential has not been fully elucidated. Nevertheless, there is definitely a strong connection between ferroptosis and immunomodulation. For instance, PD-L1-mediated immunotherapy on B16 cells enhanced CD8^+^ T cells, which in turn promoted lipid peroxidation and ferroptosis *via* INF-ɣ (96). But the inherent potential of ferroptosis as an anti-cancer immunotherapeutic agent is still under investigation. It is presumed that, like apoptosis or necroptosis, ferroptosis activates ICD by exploiting a pattern of events resembling those in other regulated cell death types, *i.e*. activation of DAMPs and release of cytokines (97, 98). Krysko’s research team was the first who was able to confirm this hypothesis (12). The authors demonstrated that murine fibrosarcoma MCA205 ferroptotic cells promote the phenotypic maturation of BMDCs and elicit a vaccination-like effect *in vivo*, proving the ability of ferroptosis to induce an adaptive immune response (**Figure 2**). ATP and HMGB-1 have proven to be the key DAMPs mediating the immunogenicity of ferroptosis (99, 100). The immunogenic nature of ferroptotic cancer cells has been recently confirmed also by other research groups. It has been identified that the inflammatory and immune response elicited by ferroptosis involves decorin as an essential DAMP that acts on the receptor advanced glycosylation end-product-specific receptor on macrophages to trigger the production of pro-inflammatory cytokines (98). Notably that using the same murine tumor prophylactic vaccination model as was used by Krysko’s team (12) but using another cancer cell line (*i.e.*, pancreatic ductal adenocarcinoma KPC cells), the authors confirmed the immunogenic potential of ferroptotic cancer cells *in vivo*. Recently, by using another murine tumor model it has been shown that arachidonic acid and IFNɣ coordinately induce tumor cell ferroptosis *via* ACSL4 and ACSL4-mediated immunogenic tumor ferroptosis (101). Of interest, other evidence of ferroptosis immunogenicity is found in non-tumor models. For example, ferroptotic cells can attract neutrophils in a model of heart injury. They do so by activating the pathway of Toll-like receptor 4 (TLR4)-TIR domain containing adaptor inducing interferon beta (TRIF), corroborating the hypothesis implicating DAMPs in ferroptosis-mediated immunity (102). All these studies strongly confirm the hypothesis of the immunogenic potential of ferroptosis and point to the need to develop new therapeutic strategies for cancers based on induction of ferroptosis.

To facilitate ferroptosis and lessen therapy-related toxicity, several nanotherapeutic models have been designed and optimized. Different types of strategies have been developed using different nanocarriers (**Figure 1**). For instance, arginine-rich silicate-nanobubbles (AMSNs) have been built to inactivate GPX4 activity through GSH scavenger activity. The arginine delivers AMSN into the tumor while the manganese silicate moiety reacts with GSH, reducing its scavenging ability and inducing ferroptosis and tumor suppression in Huh7 xenografted Balb/c mice (70) (**Table 1**). Another strategy is to offer tumor cells an apparatus that favors the Fenton reaction by catalyzing the reaction or by intratumor delivery of reagents. The Fenton reaction takes place between intratumor hydrogen peroxide and ferric ions and leads to the formation of hydroxyl radicals, thereby causing lethal lipid peroxidation (103, 104). Numerous nanocatalysts that have been developed successfully triggered ferroptosis in cancer cells. The simplest is a nanocatalyst consisting of a single PEGylated iron atom (PLGA). In the acidic tumor microenvironment, the iron is released and can support the formation of lipid peroxides, which subsequently induce both ferroptosis and apoptosis (43). Besides, hydrogen peroxide can be inserted into a Fe_3_O_4_-PLGA nanocarrier and transported into the tumor. By using ultrasound, the system is disrupted, and the liberated reagents are set free to proceed with the Fenton reaction and lipid peroxidation (42). Another sophisticated nanocarrier involves the release of iron. Briefly, an amorphous calcium carbonate nanostructure is loaded with the proapoptotic agent, doxorubicin, chelated to Fe^2+^ (Fe^2^- doxorubicin core) and coated with a complex structure that guarantees the entrance of the intact core into the cells. There, Fe^2+^ triggers ferroptosis, which in turn is synergized by the oxidative stress induced by doxorubicin. In nude mice bearing 4T1 and A375 cells, this approach efficiently inhibited tumor growth and with a very safe profile (71) (**Table 1**). Besides, iron can be targeted into tumor cells by loading a magnetosome with Fe_3_O_4_ nanoclusters. On melanoma B16F10-xenografted mice, this system was able to accumulate in the tumor site, promote tumor suppression and the generation of an immunogenic tumor microenvironment. In particular, it ameliorated the CD4^+^ T/T_reg_ cells, CD8^+^ T/T_reg_ cells, and M1/M2 macrophages *ratios* (105).

Many other nano and micro drug delivery systems efficiently induce antitumor activity by inducing ferroptosis, but their immunogenic potential has not been thoroughly investigated. It will be interesting to understand if the specific target triggered to induce ferroptosis is crucial to ensure activation of the immune system and if some nanomaterials provide stronger activation of the immune system than others. Thus, the next step is to assess if and which nanoparticles induce immunogenic ferroptosis. If, unexpectedly, nano-induced ferroptosis turns out to be not immunogenic, other strategies could be tried to create the strong antitumor and immunogenic response (listed in the next section).

## Interplay Between Nanomaterials and the Clearance of Dying Cancer Cells as a Strategy to Increase Their Immunogenicity

Recognition and efferocytosis of dying cancer cells (*i.e.*, phagocytosis) determines the subsequent immunological effects (tolerogenic, neutral, or immunogenic) (18, 106). At the two extremes, tolerogenic phagocytosis leads to active immunosuppression, while immunogenic phagocytosis promotes immunostimulatory clearance of cancer cell corpses (107). The balance between pro- and anti-inflammatory *stimuli* released from both dying and immune cells in the tumor microenvironment (TME) dictate the fate of dying cells and the immunogenicity of the outcome. Also, the type of immune response mediators (immunostimulant or immunosuppressive) decides whether the TME will be immunogenic or not. For instance, neutrophils or M2 macrophages secreting the anti-inflammatory IL-10 and TGF-β promote tolerogenic phagocytosis, while inflammatory monocytes or DCs induce an immunogenic outcome (25). Phagocytosis passes through different phases: attraction, recognition, engulfment, and processing (106). Every step is critical to resolving the immunogenic outcome and can be modulated to increase the immunogenicity of dying cells (**Figure 1**).

**Figure 4:**
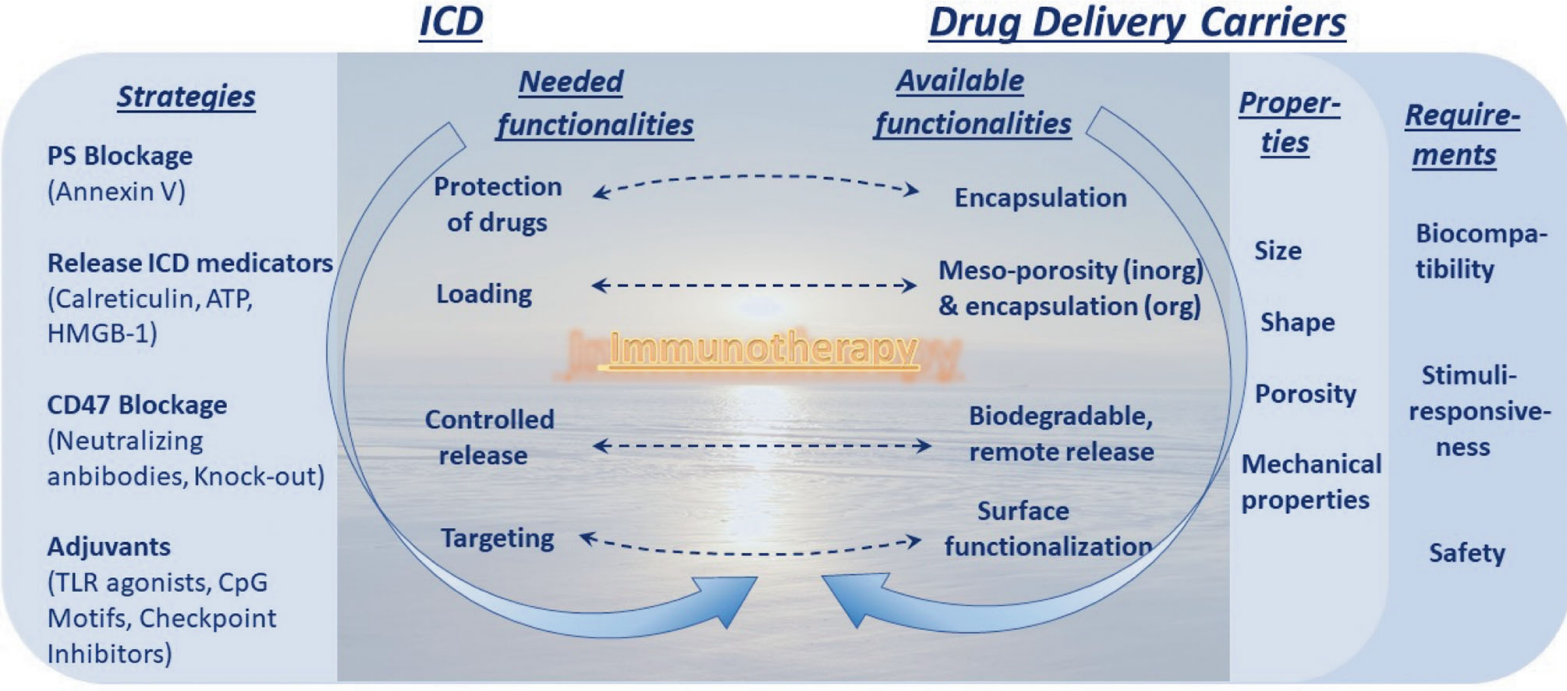
Dawn of immunotherapy spurred by immunogenic cell death (ICD) and enables by drug delivery carriers. Strategies to increase ICD and its requirements, the left column, are to be met by capabilities in the area of drug delivery carriers, the right column. This truly represents a dawn of a new era linked to immunotherapy (and this message is conveyed in the background).

One promising strategy to increase the immunogenicity of dying cells is blocking PS exposure during the early phases of cell death. For the best characterized regulated types of cell death, including apoptosis, necroptosis and pyroptosis, it has been ascertained that PS translocation to the outer side of the cell membrane acts as an early-stage “*eat-me*” signal (10, 108, 109). In each cell death type, the exposure mechanism is different, and its function can be slightly diverse. During PS exposure, necroptotic and pyroptotic cells release, respectively, proinflammatory DAMPs and cytokines such as IL-33 or IL-1β (108, 110, 111). PS-exposing necroptotic cells also cross-prime CD8^+^ T lymphocytes in a PS-dependent way. Thus, in these two cell death types, the PS exposure interval represents a window of opportunity to promote inflammation. Therefore, prolonging the time during which dying cells expose PS and blocking PS-mediated efferocytosis represents an interesting strategy to favor the development of an immunogenic TME (**Figure 4**). PS blocking also increases the immunogenicity of apoptotic cancer cells. During apoptosis, the removal of the PS “eat-me” signal impedes the phagocytosis of apoptotic cells and pushes early apoptotic cells through secondary necrosis, switching apoptosis to a proinflammatory ICD (112). The loss of the cell membrane due to secondary necrosis increases the possibility of DCs taking up tumor antigen epitopes. Consequently, blocking PS might be an appealing strategy to modulate the immunogenicity of dying cancer cells (112).

The best-known PS ligand is Annexin-V (Ann-V), which can be used to block PS and hide dying cells from phagocytosis. In practice, Ann-V disrupts PS-dependent recognition of apoptotic dying cells by macrophages both *in vivo* and *in vitro* (51), but it does not affect the uptake by immature DCs and therefore induces CD8^+^ T cell-mediated immunogenic apoptosis (112, 113), confirming the pro-immunogenicity of this strategy. Indeed, it has been shown that shielding PS by Ann-V on the surface of apoptotic dying cancer cells is an attractive strategy for increasing the immunogenicity of dying cancer cells (113). However, as the exposure time of PS is short, it is difficult to catch the right moment to use Ann-V. Nano-formulations proved to overcome this inconvenience by co-delivery of an apoptosis-inducing agent and Ann-V, allowing on-demand release. In this way, optimal PS blocking was achieved on cue, maximizing the immunogenic potential. In a recent study, Ann-V was immobilized in A diselenide-bridged, hollow, mesoporous, organosilica nanocapsule to be released by biodegradation in the oxidative TME or reductive intracellular milieu. Apoptosis was induced in 4T1-bearing Balb/c mice by laser irradiation during Ann-V release. As above, macrophage phagocytic clearance was blocked and M2 macrophage levels decreased, while the phagocytosis of these apoptotic cells by DCs was not affected. Moreover, HMGB-1 release was increased, while myeloid-derived suppressor cells and immunosuppressor Treg cells decreased, with the consequence of favoring DC maturation and activation and boosting the INF-ɣ secreting-CD8^+^ T response. The overall effect was tumor regression in half of the mice. In other words, Ann-V shifted the apoptotic cell clearing program from tolerogenic to immunogenic, with a significant improvement in the antitumor antigen-specific cytotoxic T cell immune response (112), indicating that this strategy is very promising for increasing dying cell immunogenicity (**Figure 4**). Though this method has not been applied to cancer cells undergoing other modalities of cell death, these results portend a wide application of this strategy.

Since the balance between “*eat-me*” and “*don’t eat me*” signals defines whether dying cells will promote immunogenic or tolerogenic cell death, another strategy to improve the chances of stimulating the immune system is neutralization of negative mediators of efferocytosis. CD47 is a don’t eat-me signal and immune checkpoint inhibitor (21, 114, 115). The use of specific antibodies to disable CD47 significantly increased the recognition of dying cells by the immune system and its activation. Antigen presentation by DCs was improved, together with macrophage-mediated clearance of tumor cells (116–120). Furthermore, vaccination with tumor cells lacking CD47 or pre-coated with anti-CD47 antibodies triggered T cell-mediated antitumor responses (121). Furthermore, nanomaterials could be exploited for delivery of neutralizing antibodies as an intermediary in anti-CD47-related immune therapy. Besides that, several adjuvants can boost and drive immunity by promoting local inflammation and cytokine production, activating antigen-presenting cells, or increasing the magnitude and function of the T cell response (107). Among the many adjuvants, the ability of TLR agonists, CpG motifs and check-point inhibitors to boost the immunogenicity of dying cells has been also documented (**Figure 4** **)**.

A different strategy to increase the immunogenicity of dying cells could emerge from deeper knowledge of the main ICD mediators, DAMPs. The molecular nature and the spatial-temporal frame in which these immunostimulant elements are presented to the immune system are indeed two critical factors in ICD induction (6, 25). For this reason, an ambition could be to engineer nanoparticles capable of delivering different compounds in a time-dependent way in order to deliver different “*find-me*” and “*eat-me*” factors in the same order that prompts ICD. It is well documented that the effectiveness of DAMPs requires release in a specific order, with CRT exposure being an early signal preceding even PS translocation, followed by ATP and HMGB-1 release (6, 12, 25). It is conceivable that the surface of nanoparticles could be loaded with ATP, and HMGB-1 could be conjugated with CRT to mimic, within the tumor, the appropriate spatial-temporal pattern of DAMPs release required to trigger ICD. In this way, nanoparticles would immediately expose CRT and then release ATP and HMGB-1 (**Figure 4**).

Taking all these notions together, it is evident that many strategies can be exploited to boost the immunogenicity of dying cells, and that they represent a concrete scope for improvement over the current immune anticancer therapy based on modulating the immunogenicity of dying cancer cells (**Figure 4**). By supporting cell death induction and increasing the overall immunogenic effect, nanoparticles make it possible to overcome every shortcoming of every type of cell death that usually limits its therapeutic use. The increased anticancer immune-efficiency of nanoparticle-mediated cell death is also due to the specificity of such vectors for tumor cells and the consequent limitation of toxicity. The lower toxicity could also be due to the possibility of lowering the doses of the anticancer agents because nanoparticles can boost the drugs’ potency. Although further pre-clinical and clinical studies will be needed to bring nano-mediated cancer vaccines from the bench to the bedside, the use of nanoparticles to boost the anticancer immunogenicity of dying cancer cells seems to raise the benefit/risk ratio of such therapies considerably, which indicates that this is a promising approach to pursue.

## Conclusions and Final Remarks

Induction of ICD is a promising strategy for triggering effective anti-tumor immune responses. However, only a limited number of anti-cancer therapies are available to induce ICD in experimental cancer therapies and even much less is available for clinical use. Several different nanomaterials can be tailored to target specific immunological patterns in order to promote immunogenic apoptosis, necroptosis, ferroptosis or pyroptosis, or to synergize with other immunotherapies, such as checkpoint inhibition. Of note that drug delivery carriers can be used to increase the immunogenicity of cancer cells killed by anti-cancer therapy. They can be used at least at two different levels to increase immunogenicity of dying cancer cells (**Figure 4**). The first level is based on induction of a particular type of ICD which will allow to overcome often occurring in tumor cells resistance to a particular cell death type. The second level is based on modulation of the uptake (i.e., efferocytosis or phagocytosis) of dying cancer cells because recognition and phagocytosis of dying cancer cells determines the subsequent immunological effects to be either tolerogenic, neutral, or immunogenic and thus represent a critical decision point for mounting of an efficient anti-cancer immunity. These strategies allow to convert immuno-suppressive into immunostimulatory cancer cell death and such approach can be used for cell-based therapy *in situ* (therapeutic vaccination) to enhance anti-tumor immune responses.

Although pre-clinical studies on the use of nanomaterials to promote ICD have been conducted (122) and some are on-going, essential challenges exist in pre-clinical studies on nanomaterials and in their clinical translation (123, 124). One general yet essential requirement in clinical application is ensuring the safety of nanomaterials for patients, especially for any new class of materials. The seeming discrepancy between published research and approved drugs is explained by the substantial time it takes to complete safety trials and obtain clearance (125). Reflecting on the necessity of good regulation of nanomaterial safety, regulatory measures have been adopted by the FDA in the USA (126) and regulatory commissions in EU (127, 128). Indeed, the future perspectives are closely linked with on-going clinical trials, of which approximately 98% of about 379 trials are related to apoptosis. Taking into consideration that the other cell death modalities were discovered much later than apoptosis, this is not surprising. There are no clinical trials on ferroptosis while only a few clinical trials on necroptosis and pyroptosis. The safety of the newly introduced materials also remains a vital issue for performing pre-clinical and clinical trials on the application of ICD (129, 130). Further progress in this area is also linked with the possibility of determining which nanomaterials induce which cell death modalities, where new approaches are needed. Briefly, we can summarize the challenges for the application of nanomaterials to increase the immunogenicity of dying (dead) cancer cells for anti-cancer therapy as follow:

- It is important to be able to control the immunogenicity of nanomaterials. In this context, PLGA has been shown to be a suitable material (131), and efforts are being invested in extending the scope of new immunogenically relevant materials. Immunogenic peptides that can be presented by MHC Class I and II complexes for interaction with the T cell receptor (TCR) were recently reported (132).
- It is essential for nanomaterials to be biocompatible and, if necessary, biodegradable. Emphasizing these important attributes, a number of both synthetic and natural molecules can be used to modulate ICD (133). Another important aspect of the use of nanomaterials lies in combining the best properties of various constituents of nano-carriers (134). An interesting way to accomplish that could be by combining the organic and inorganic nanomaterials, as recently reported (135).
- More insight is needed into how the size and the surface properties of nanomaterials (charge, functionality) (136) influence their interaction with dying cancer cells. Although the size of nanoparticles affects their uptake by cells (137), limited data are available on the correlation between the size of nanoparticles administered at realistic doses (138) and clinically relevant toxicity patterns (139).
- Novel nanomaterials have been developed and applied for antigen presentation relevant for immunogenic cell death. For example, polyelectrolyte microparticles, which can be controlled by various external actions (140) and have advanced release capabilities (141), have been reported to be effective enhancer of antigen cross-presentation in porcine dendritic cells (142). Further, lipid-based formulations (143, 144), anisotropic (145), multicompartment (146), anisotropic multicompartment (147) carriers, soft and hard nanocarriers (148) are some of the candidates for incorporating advanced functionalities for immunology.
- Among different types of carriers, theragnostic drug delivery vehicles (149) are seen to be a particularly valuable type of carriers due to their dual function: diagnostic or sensoric (150) as well as therapeutic functions.
- Sufficient statistically significant data must be ensured in two adjacent areas. In biology and medicine, it is difficult to obtain sufficiently extensive statistical data from animal experiments and even more difficult from humans. Furthermore, if this approach is to be translated to the clinic, it is necessary to take into account the variability between patients, which incurs substantial time and financial costs. In addition, to assure broad application, medically relevant data need to be collected for sufficiently diverse populations. In nanotechnology, extensive efforts have been recently invested in addressing transparency and reproducibility, and a minimum information standard has been launched for reporting experimentation on bio−nano interactions in bio-nano experimental literature (MIRABEL) (151).
- To launch and implement clinical trials, it is vital to provide effective feedback from those trials to scientists developing novel materials for modulation of ICD. Moreover, the ethics of animal experimentation also have to be considered, particularly in view of the new regulations (152) that introduced new restrictions on the use of animals.

But despite all these challenges discussed, solid progress has already been achieved. This progress can be seen when examining examples of the application of photodynamic therapy based on novel photosensitizers (153, 154) and employing new materials and approaches to modulate the immunogenicity of dying cancer cells (**Table 1**). Various approaches and materials have been developed to target several cell death modalities including apoptosis, necroptosis, pyroptosis and ferroptosis. One example of such development is death-inducing gene therapy by using plasmid DNA (155), for which there is mounting preclinical evidence for its potential clinical application (156). Another development is repurposing nanoparticles for drug delivery to enhance ICD responses (157). Furthermore, nanoparticles with specific functions, for example, iron-catalysis, have been shown to induce ferroptosis (158).

The most significant hurdle to the implementation of nanotechnology and nanomedicine (159) in immunotherapy (i.e., in ICD) is defining the targets that result in the most effective immune response. Indeed, to improve the design of nanomaterials, deeper knowledge of the mechanisms of ICD is needed. Therefore, ICD basic research should go hand-in-hand with the development of nanoparticle-based therapeutics (**Figure 4**) which is largely driven by the availability of new nanomaterials. In the future new technologies are needed to be developed to distinguish different ICD modalities not only *in vitro* but also in *in vivo* context (50, 87, 160). In conclusion, interdisciplinarity, interconnectivity and interdependence of immuno-oncology on the one hand and nanomaterials and nanotechnology on the other hand are expected to drive further progress in the use of nanomaterial in anti-cancer therapy based on ICD induction.

## Author Contributions

EC, OF, AS, and DK designed the manuscript and recommended a structure for the review. EC, AS, and DK wrote the initial draft and prepared the figures. All authors contributed to the article and approved the submitted version.

## Funding

DK lab is supported by FWO-Flanders (G016221N). AS and DK acknowledge the support of FWO-Flanders (G043219N) and Ghent University BOF (Special Research Fund; IOP 01/O3618). AS acknowledges support of FWO-Flanders (I002620N) and BOF (Special Research Fund (BAS094-18, BOF14/IOP/003). This study is also supported by the Ministry of Science and Higher Education of the Russian Federation, agreement No. 075-15-2020-808. This project (40007488) has received funding from the FWO and F.R.S.-FNRS under the Excellence of Science (EOS) program to OF, AS, and DK.

## Conflict of Interest

The authors declare that the research was conducted in the absence of any commercial or financial relationships that could be construed as a potential conflict of interest.

## Publisher’s Note

All claims expressed in this article are solely those of the authors and do not necessarily represent those of their affiliated organizations, or those of the publisher, the editors and the reviewers. Any product that may be evaluated in this article, or claim that may be made by its manufacturer, is not guaranteed or endorsed by the publisher.
